# Progress and Prospects of Bioelectrochemical Systems: Electron Transfer and Its Applications in the Microbial Metabolism

**DOI:** 10.3389/fbioe.2020.00010

**Published:** 2020-01-31

**Authors:** Tianwen Zheng, Jin Li, Yaliang Ji, Wenming Zhang, Yan Fang, Fengxue Xin, Weiliang Dong, Ping Wei, Jiangfeng Ma, Min Jiang

**Affiliations:** State Key Laboratory of Materials-Oriented Chemical Engineering, College of Biotechnology and Pharmaceutical Engineering, Nanjing Tech University, Nanjing, China

**Keywords:** bioelectrochemical system, electron transfer, microbial fuel cells, microbial electrolysis cells, energy generation, coenzyme metabolism

## Abstract

Bioelectrochemical systems are revolutionary new bioengineering technologies which integrate microorganisms or enzymes with the electrochemical method to improve the reducing or oxidizing metabolism. Generally, the bioelectrochemical systems show the processes referring to electrical power generation or achieving the reducing reaction with a certain potential poised by means of electron transfer between the electron acceptor and electron donor. Researchers have focused on the selection and optimization of the electrode materials, design of electrochemical device, and screening of electrochemically active or inactive model microorganisms. Notably, all these means and studies are related to electron transfer: efflux and consumption. Thus, here we introduce the basic concepts of bioelectrochemical systems, and elaborate on the extracellular and intracellular electron transfer, and the hypothetical electron transfer mechanism. Also, intracellular energy generation and coenzyme metabolism along with electron transfer are analyzed. Finally, the applications of bioelectrochemical systems and the prospect of microbial electrochemical technologies are discussed.

## Introduction

Bioelectrochemical systems, revolutionary new bioengineering technologies, integrate microorganisms or other bio-based catalysts with an electrochemical method to improve the reducing or oxidizing metabolism. Generally, bioelectrochemical systems show the process of electrical power generation or achieve the reduction reaction with a certain potential poised by means of electron transfer between the electron acceptor and electron donor (Fernandez et al., 2015).

Previously, bioelectrochemical systems have been widely applied in the form of microbial fuel cells (MFCs), since Michael Potter (1911) first studied the generation of an electrical current by several microorganisms, which convert chemical energy into electrical energy by degradation of various substrates, especially organic compounds from waste water (Gul and Ahmad, 2019). As a reversal process compared to MFCs, microbial electrolytic cells (MECs) were used to convert electrical energy to chemical energy with the help of microorganisms or enzymes to produce useful products such as formate, methanol, ethanol, or hydrocarbons. These molecules were then converted or used directly as a sustainable alternative to fossil fuels (Yuan et al., 2019). With the growing desire for environment-friendly and energy-saving processes (Zhang and Angelidaki, 2015), bioelectrochemical systems attracted much attention for their green and sustainable characteristics (Ghosh and Ghangrekar, 2015).

To expand the application of bioelectrochemical systems, researchers have focused on the selection and optimization of the electrode materials, design of the electrochemical devices, and screening of electrochemically active or inactive model microorganisms. Recently, bioelectrochemical systems have been widely applied in nitrate removal, solid waste processing, desalination and materials science (Zhen et al., 2016). Notably, all of these uses and studies are related to electron transfer and energy transformation, i.e., the interchange of chemical energy and electrical energy.

Here, we first introduce the basic concepts of bioelectrochemical systems, and elaborate on the mechanisms of extracellular and intracellular electron transfer. We also analyze the hypothetical electron transfer mechanism, intracellular energy generation and coenzyme metabolism. Finally, the applications of bioelectrochemical systems and prospective microbial electrochemical technologies are discussed.

## Classification of Bioelectrochemical Systems

An electrochemical system is a series of electrochemical models, or devices, in which electronic behaviors occur with different types of catalysts. In terms of bioelectrochemical systems, it mainly describes a series of technologies that are used for biotechnology applications including electricity generation (Butler et al., 2010) and the production of valuable products (Villano et al., 2011). Generally, they can be divided into two categories depending on the catalyst adopted. The first one are those that use microorganisms as catalysts, and the second are those that use enzymes as the catalyst.

### Microbial Electrochemical Systems

According to the direction of electron transfer and the type of reaction, microbial electrochemical systems can be divided into MFCs and microbial electrolysis or electro-synthesis cells (MECs). MFCs, are electrochemical systems with microorganisms acting as biocatalysts in the anode chamber, have been widely used for electricity generation with various substrates (Table 1). In MFCs, electrons released through intracellular metabolism (substrate oxidation) transfer to the anode, and are finally captured by the cathode electrode *via* an external circuit to be used for the reduction of oxygen, or another electron accepter, with the generation of current (Logan et al., 2006). Recently, many MFCs have been reported that act as an innovative wastewater treatment technique for pollution removal and energy generation, due to their high degradation rate (He et al., 2015; Yazdi et al., 2015; Zhao et al., 2015a).

**TABLE 1:** Brief summary of microbial fuel cells.

**Strains**	**Anode electrode**	**Cathode electrode**	**Substrate**	**Electrode surface (cm^2^)**	**Electron shuttles**	**Power output**	**References**
Anaerobic sludge	Graphite felt	Graphite felt	Glucose	10	NA^a^	28.6 mW/m^2^	Song et al., 2016
Anaerobic sludge	Activated carbon cloth	Carbon cloth	Waste water	66.5	NA	142 mW/m^2^	Yazdi et al., 2015
Activated sludge	Modified carbon cloth	Modified carbon cloth	Sodium acetate	2.5	NA	2355 mW/m^2^	Yuan et al., 2016
Activated sludge	Carbon brush	Bilirubin oxidase	Acetate	9	NA	6530 mW/m^2^	Santoro et al., 2016
Consortium	Ammonia-treated carbon cloth	Carbon fiber	Cellulose	1.13	NA	5.4 mW/m^2^	Farzaneh et al., 2009
*Geobacter* biofilm	Modified graphite rod	Modified graphite rod	Acetate	5.81	NA	100 mW/m^2^	Commault et al., 2015
*Geobacter sulfurreducens Escherichia coli*	PTEE carbon cloth	PTEE carbon cloth	Acetate	7	NA	9.8 mW/m^2^	Qu et al., 2012
*Shewanella oneidensis*	CP/G/Au	CP/G/Au	Lactate	6	-	508 mW/m^2^	Zhao C. et al., 2015
*Spartina anglica*	Plant root	Plant root	Waste water	27	NA	679 mW/m^2^	Wetser et al., 2015
Recombinant consortium	Carbon cloth	Carbon cloth	Glucose Xylose	6.25	Flavins	104.7 mW/m^2^	Li et al., 2019
*Shewanella oneidensis*	Carbon cloth	Carbon cloth	Lactate	1	Flavins	2630 mW/m^2^	Lin et al., 2018

*^*a*^No electron shuttles added in electrochemical system.*

Bioelectrochemical systems have been successfully applied in MFCs using diverse microorganisms such as *Shewanella putrefaciens* (Yang et al., 2017), *Shewanella oneidensis* MR-1 (Bretschger et al., 2007) and *Escherichia coli* DH5α (Li et al., 2018), which can produce a maximum power density range from 3800 to 4400 mW/m^2^. However, these fuel cells have a low cost-effectiveness due to high material costs and mediocre power generation, which are still the major limitations to extending the range of applications (Zhou et al., 2013). Also, some electrodes are manufactured with gold, platinum and other expensive materials which raises the cost (Sadeghifar and Rashid-Nadimi, 2017).

Microbial fuel cells systems adopt bacteria as catalysts, and the biofilm is formed on the surface of the anode, which can improve the electron transfer. Thus, the practical application of MFCs may be further confined by microorganisms - since the efficiency and stability of microbial reactions mainly relies on the external environment which is far different from their indigenous environments (Pareek et al., 2019), due to issues such as low temperatures, high salinity and high toxicity (Zhang et al., 2017). Another limitation is the internal resistance of the fuel cell caused mainly by the proton exchange membrane, which results in low current density and power density (Shreeram et al., 2018). Table 1 lists the recent research on MFCs.

Microbial electrolytic cells also use microorganisms as catalysts, and the cells are inoculated into the cathode chamber which acts as an electron acceptor and gains electrons, thus accelerating the intracellular reduction metabolism (Villano et al., 2011; Ding et al., 2012; Jafary et al., 2015). Essentially, MECs are a reverse process compared to MFCs. In MFCs, the oxidation reaction occurs in the anode chamber, after which the released electrons are transferred to the cathode chamber in a process that involves substrate reduction. For MECs, the potential poised on the cathode chamber is the most important factor and is determined by the bacteria and electron shuttles added, as they can have different potential differences (Table 2). However, the potential poised on the cathode is not equal to the theoretical potential difference of electron shuttles as the electron or energy losses in bioelectrochemical system (Zhen et al., 2016). There are also many issues restraining the scale up of MECs, especially as most of the microbial electrosynthesis systems suffer from low energy efficiencies and low production rates (Su and Ajo-Franklin, 2019). Another limitation on MECs is chemical incompatibility between the abiotic and biotic catalysis, for example, fouling of the electrode by microbes, or toxicity to the microbes caused by electrode leaching (Gildemyn et al., 2017). A vital index in the application of MECs is the long-term stability, which suffers from low turnover frequencies and oxygen sensitivity of certain enzymes relevant to CO_2_ reduction (Yuan et al., 2019).

**TABLE 2:** The standard potential of electrons shuttles.

**Electron shuttles**	**Standard potential (*E*^0′^/*V*)**	**Electron mediators**	**Standard potential (*E*^0′^/*V*)**
Methyl viologen	−0.446	NAD^+^/NADH	−0.315
H_2_	−0.414	Methane/HCO_3_^–^	−0.24
Neutral red	−0.325	FAD/FADH2	−0.219
Riboflavin	−0.208	MK/MKH_2_	−0.074
Anthraquinone-2,6-disulfonate (AQDS)	−0.184	Fumarate/Succinate	+0.031

By using cyclic voltammogram detection, it was found that the potential poised cathode can drive the electron shuttle reduction at high rates and then the intracellular reducing metabolism can be accomplished by current stimulation. During the whole process, the cathode provides electrons to pump intracellular reducing power and energy generation (She et al., 2006). However, it remains to be investigated whether the electrons released from the cathode can be efficiently transferred between the cathode and bacteria (Schroder, 2007). In a word, in these two microbial electrochemical systems, the possibility of electron transfer and the rate of electron transfer between cells and electrodes are the key factors used to determine the efficiency of the entire system (Li et al., 2015; Santoro et al., 2015).

### Enzymatic Electrochemical Systems

Another type of bioelectrochemical system is that using enzymes as catalysts. The electrodes with enzymes serve as external electron donors or electron acceptors (Amano et al., 2016; Lee et al., 2016). Since the enzymatic reaction is the sole reaction occurring in this electrochemical system, and the electron transfer kinetic potential is predetermined, and the oxidoreductase can be regenerated by capturing or releasing electrons at the surface of electrode, the enzymatic electrochemical system is widely used in the study of electron transfer mechanisms *in vitro* (Kracher et al., 2016).

Since previous studies have applied electrochemical methods to study the biological electron and ion transfer and proved its high sensitivity and reliability, it would be an efficient and convenient strategy to analyze the mechanism of enzyme reaction. When NADH:quinone complex I was fixed on the surface of a gold modified electrode, the process of electron and proton transfer recurred effectively *in vivo* with the electrode as the sole electron acceptor (Oscar et al., 2014). Moreover, in order to increase the load of the enzyme for a higher catalytic rate, high surface area materials were introduced into this system with a high density current output (Bari et al., 2016).

Although all these electrochemical systems have been studied for decades, upgraded electrochemical devices and novel biocompatible electrode materials are absolutely imperative. Moreover, the mechanisms of energy output and electron transfer are still in their infancy.

## Electron Transfer in the Bioelectrochemical System

### Extracellular Electron Transfer

#### Electron Transfer in Enzyme Electrochemical Systems

In enzyme electrochemical systems, the oxidoreductases are selected, purified and fixed on the surface of modified electrodes, which act as electron donors or electron acceptors and participate in enzymatic reactions along with the interaction of an electrode (Compagnone and Guilbault, 1997; Bari et al., 2016). So, the key problem to be solved is the bidirectional electron transfer from the electrode to the active site of the enzymes. However, the transition and transfer of electrons between the electron carriers have certain restrictions, as the relative distance between the two given electron carriers within the enzyme increases, the electron transfer rate will decline rapidly and affect the efficiency of the enzymatic reaction. When the distance is longer than 10 angstroms, electron transfer will only be achieved through the presence of electron mediators (Mayo et al., 1986). Therefore, the electrodes are modified to facilitate the immobilization of the enzyme and thus effectively accelerate electron transfer between oxidases and the electrodes (Freguia et al., 2012; Oscar et al., 2014). Furthermore, conductive nano-particles can also be used to assist the long distance transfer of electrons. In this way, a long distance electron transfer from FAD/FADH_2_ (glucose oxidase) to an electrode was achieved (Degani and Heller, 1987).

Although efficient electron transfer can be achieved by various means, the applications of enzyme electrochemical are restricted to a small field by taking into consideration the decreasing activity of enzymes during recycling. Among them, enzyme electrochemical sensor systems is a relatively mature field and widely used for substance detection, such as heavy metals (Ruecha et al., 2015), glucose (Gutierrez et al., 2016) and other organic substances (Wang et al., 2016). Additionally, enzyme electrochemical systems have also been successfully applied to studying the mechanism of electron transfer during enzyme reactions. By cyclic voltammetry detection, Kracher et al. (2016) verified the mechanism of electron transfer in lytic polysaccharide monooxygenases (LPMOs) during the oxidative degradation of cellulose with multi-enzyme modified electrodes.

#### Extracellular Electron Transfer in Microbial Electrochemical Systems

**FIGURE 1:**
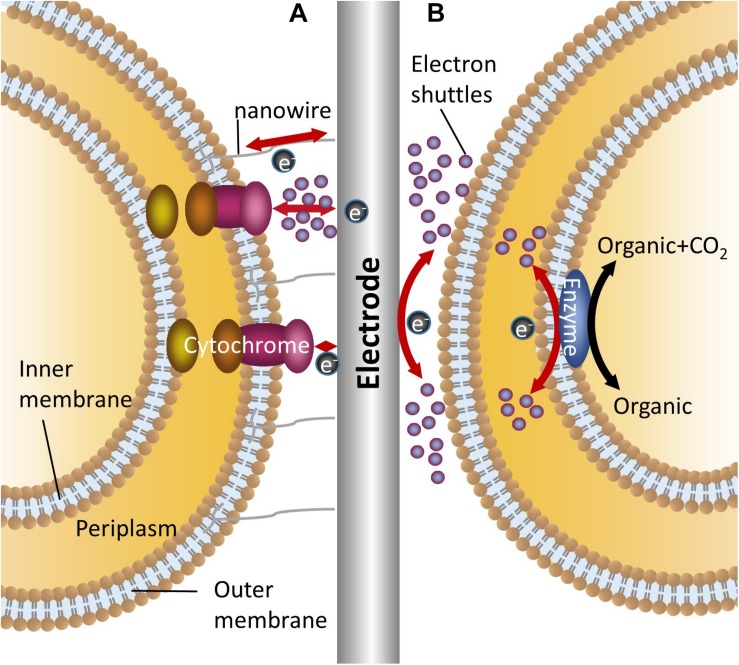
Mechanisms for bidirectional electron transfer between bacteria and electrodes. **(A)** Represents two mechanisms of direct electron transfer, one is mediated by nanowire, the other is mediated by outer membrane cytochromes with or without electron shuttles; **(B)** Shows the indirect electron transfer mediated by electron shuttles.

Unlike enzyme electrochemical systems, microorganisms act as catalysts in MFCs and MECs. In MFCs, reducing equivalents stored in the organic substrate are released in the form of electrons, which are captured by the anode and then transferred to the cathode through the external circuit with the generation of electricity (Santoro et al., 2016). In MECs, a given voltage is poised at the cathode. The electrons involved in intracellular reduction and energy metabolism are released from the cathode electrode and captured by strains (Carmona-Martínez et al., 2015; Yin et al., 2016). Since the cell membrane is insulated, a necessary prerequisite for the electrochemical reactions to occur is that the electrons can smoothly transfer across the membrane (Kracher et al., 2016). Due to reactions occurring on the electrodes of different substances exhibiting different electrochemical behaviors and the different mechanisms of electron transfer between microorganisms and electrodes, three major mechanisms exist in electron transfer between the electrodes and the microorganisms (Figure 1). They are reling on nanowires (conductive pili), relying on outer membrane proteins and/or mediated by endogenous or exogenous electron shuttles (Choi and Sang, 2016).

For the electrochemical active strains, different groups of strains evolved different conductive mechanisms. *Shewanella* has a complete set of extracellular electron transport chains. The electron transport channels which were formed by outer membrane cytochromes (Mtr and CymA system) mediate the direct electron transfer between cells and electrodes (Ross et al., 2012; Carmona-Martínez et al., 2013). Meanwhile, the synthesis and release of riboflavin assists electron transmembrane transfer, but this process requires a specific transport system (Brutinel and Gralnick, 2012). Another model electrochemical active strain, *Geobacter*, can synthesize conductive pili (nanowires) through cell growth and is directed involved in the extracellular electron transfer between cell and electrodes (Schroder, 2007; Lovley, 2008). Also, outer membrane cytochromes are necessary for electron capturing (Butler et al., 2010).

Nanowires, outer membrane cytochromes and other appendages are key components for effective electron capture in electrochemical active strains. In order to improve the extracellular electron transfer efficiency, the development of biofilms can benefit the whole process (Verea et al., 2014; Laura et al., 2015). The presence of biofilm ensures direct long distance electron transfer, high catalytic rates and high power outputs. In recent years, newly developed and modified porous conductive materials have been used for facilitating biofilm formation on electrode surfaces (Karthikeyan et al., 2015; Yuan et al., 2016). Although the electrodes that have porous or three-dimensional surfaces in MFCs will improve the performance of the whole system with enhanced efficiency of electricity output, it also depends on the conductive characteristic of the selected electrode material (Ledezma et al., 2015). When using stainless steel and carbon felt as electrodes for current generation, Dumas et al. (2008) found that carbon felt has high porosity characteristics for microbial attachment, but weaker conductivity compared to stainless steel electrodes which caused lower level current generation. However, in terms of other electrochemical active or inactive strains, the electrons cannot transfer directly between the cells and electrodes, and biofilm on electrode surfaces limited the electron and mass transfer (Rabaey et al., 2007; Freguia et al., 2008).

The electrochemically inactive bacteria do not have a fully functional extracellular electron transfer system and almost all the strains of this type are not able to secret electron carriers (Masuda et al., 2010). However, electrochemically inactive bacteria can react with electrodes through the addition of an electron shuttle. In the majority of model strains, such as *Escherichia coli*, *Actinobacillus succinogenes* (Park et al., 1999) and *Clostridium* (He et al., 2016), bidirectional electron transfer can be achieved in the presence of electron shuttles. With neutral red, *A. succinogenes* can gain electrons from a cathode and use these for intracellular metabolism (Park et al., 1999). Although bacteria can be divided into electrochemically active and inactive types, the boundary is not so clear. For example, one study has demonstrated that the screened *E. coli* gained the ability of direct electron transfer from intracellular to extracellular and achieved electricity generation without electron shuttles (Park et al., 1999). However, that does not mean all the electrochemically inactive bacteria can gain the same ability of direct electron transfer.

#### Interspecies Electron Transfer

After the microorganisms were incubated into electrochemical systems, a start-up period was needed to activate the process of electricity generation. During this period, electrochemically active strains, in which electrons could be transferred directly were absorbed onto the surface of the electrode to form biofilm. And thus, a shorter start-up period of MFC could be obtained as the distance of electron transfer decreased (Zhao et al., 2015b).

A co-culture of *Geobacter* and *Methanosaeta* or *Methanosarcina* species has been confirmed to promote the electro-synthesis of methane as strains shared electrons via direct interspecies electron transfer (Zheng et al., 2015). In this way, interspecies electron transfer can promote and strengthen the symbiotic electrochemical behavior with microbial co-culture fermentation, which was beneficial to achieve synergy between different microbial species and the production of bio-gas or other high value biofuels from cheap raw materials (Zheng et al., 2015).

The more general concept is that conductive materials can promote interspecies electron transfer and strengthen co-culture fermentation. In order to introduce more species to symbiotic co-culture systems and expand the application of interspecies electron transfer, electron shuttles can also be used for indirect electron transfer among strains. It is demonstrated that electron transfer mediated by active carbon particles within cells does not need to rely on cellular conductive structures (such as conductive pili or nanowires) or the assistance of cytochromes (Liu et al., 2012; Amelia-Elena et al., 2014). Moreover, the interspecific electron transfer can be achieved in the presence of activated carbon particles, and the catalytic properties of the mixed bacteria can be strengthened even when co-cultured with electrochemically inactive bacteria (Qu et al., 2012).

It is worthy of notice that co-cultures of bacteria with different electron shuttles may have different functions. In the presence of AQDS, co-cultures of *Geobacter metallireducens* and *Geobacter sulfurreducens* can achieve higher rates of ethanol consumption and better cell growth as more energy is generated (Smith et al., 2015). When the co-cultured bacteria were *Geobacter metallireducens* and *Monascus barkeri*, no obvious effects on the synthesis of methane from ethanol was obtained because of the higher potential of AQDS (Table 2; Liu et al., 2012). This can be explained due to the differing levels of intracellular energy that can be gained along with electron transfer from electron donor to electron acceptor *via* various electron shuttles and electron transfer chains.

### Intracellular Electron Transfer Chain

The only goal of nanowires, electron shuttles and modified electrodes is to strengthen the interaction between cells and electrodes, and to increase the rate of extracellular electron transfer. The intracellular electron transfer chain (ETC, also called the respiratory chain) consists of a series of electron or proton carriers, including cytochromes, coenzyme Q and lots of oxidoreductases. Within the ETC, electrons are transferred from high potential electron donor to a low potential electron acceptor along with ATP synthesis (Hara and Kondo, 2015).

**FIGURE 2:**
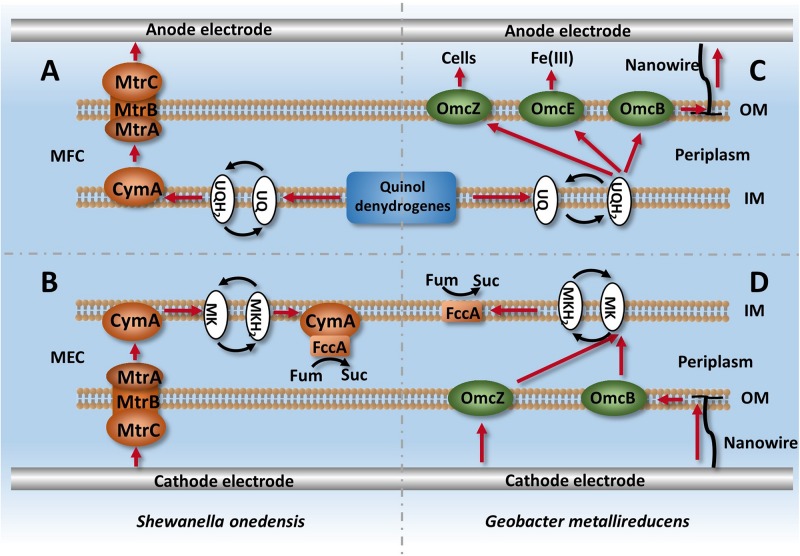
Intracellular electron transfer chains in *Shewanella* and *Geobacter*. **(A)** Describes the process of electron leaking from intracellular metabolism via Mtr and CymA systems in *Shewanella*, **(B)** Describes the process of electron capturing by strains from cathodes and used for fumarate reduction. **(C,D)** describe the ETCs of *Geobacter* in MFC and MEC systems, respectively.

For electrochemically active bacteria, cytochromes that are anchored at the membrane can facilitate electron transfer and intracellular metabolism (Richter et al., 2009). As electrochemically active bacteria, *Shewanella* and *Geobacter* have an excellent ability for intracellular electron transfer, and thus are widely used in MFCs (Lovley, 2012). Previous studies have demonstrated that some strains can change electron transfer routes depending on the potential difference of available electron donors or acceptors (Kracke et al., 2015), and *Shewanella* can achieve bidirectional electron transfer with only one intracellular electron transfer system in MFCs and MECs (Figure 2; Shi et al., 2010; Ross et al., 2012). The only difference was the electron mediator that was used. The MFC adopted ubiquinone, while the MEC adopted menaquinone, as it has a lower potential difference to ubiquinone. Compared with *Shewanella*, three electron leaking mechanisms exist in *Geobacter*: electron transfer OmcZ between cells, OmcE/OmcS used for Fe (III) reduction, and nanowire used for interactions with electrodes (Richter et al., 2009; Shi et al., 2010; Ross et al., 2012; Sturm et al., 2015).

Electrochemically inactive bacteria can interact with electrodes in the presence of electron shuttles (Table 2). Bio-based electron shuttles, such as riboflavin, coenzyme Q or its analogs, can integrate into inherent electron transfer chains directly *via* membrane transporters or diffusion, and participate in intracellular reducing and energy metabolism (Schroder, 2007). Compared with bio-based electron shuttles, chemical based electron shuttles have a faster diffusion rate, and their high polarization characteristics ensure effective bidirectional transportation across membranes (Federico et al., 2007; Pandit and Mahadevan, 2011). However, the relationship between chemical-based electron shuttles and intracellular ETC is not clear. Some studies showed that neutral red can integrate into membranes and execute the function of native electron transfer mediators (Park et al., 1999). Recently, Harrington et al. (2015) verified that neutral red can interact with menaquinone and then transfer the electrons into intracellular ETC. Another type of electron shuttles are reduced chemical substances, such as hydrogen and formate (Zhao et al., 2006).

Until now, it has been confirmed that electron shuttles can capture or release electrons between electrodes and intracellular ETC even though the mechanism is not clear. Compared with electrochemically active strains, the electron transfer efficiency of intracellular ETC in electrochemically inactive strains in not effective. Thus, metabolic engineering strategies have been applied to reconstruct the intracellular ETC to promote electron transfer in electrochemically inactive bacteria. Sturm-Richter et al. (Sturm et al., 2015) found that heterologous expression of intracellular ETC from *Shewanella* (CymA and Mtr system) in *E. coli* can reprogram the intracellular metabolism and accelerate the intracellular electron transfer rate by 183% (Sturm et al., 2015). In addition, higher electricity power output was achieved with the assistance of methylene blue. Similar results showed that the rate of extracellular electron transfer can also be increased by heterologous introduction of a synthetic flavin pathway in *Shewanella* (Yang et al., 2015).

## The Effects and Applications of Bioelectrochemical Systems in Microbial Metabolism

Electrons are not simply transferred along with the potential gradient between the electrode and cell, or by intracellular electron transfer chains in bioelectrochemical systems, and the reason for the presence of electron carriers is not just to transfer electrons by a simple pattern from an electrode to intracellular ETC. The capturing of electrons is often accompanied by the cotransport of protons (Pandit and Mahadevan, 2011), which can be released and involved in intracellular reduction and energy metabolism. In addition, the reducing power (NADH or FADH_2_) and ATP play a vital role in intracellular redox metabolism, metabolite synthesis and transportation, stress responses and transcriptional regulation (Balzer et al., 2013; Man et al., 2016). The perturbation of intracellular ATP and NADH levels has effects on the whole cellular metabolism and redirects the metabolic flux (Holm et al., 2010).

### Energy Metabolism

In bioelectrochemical systems, bacteria can gain energy in two ways. First, the native respiration chain is the main route of energy generation. For electrochemically active strains, insoluble metals act as electron acceptors and participate in extracellular ETC in nature. When *Geobacter* and *Shewanella* were inoculated in MFC system, they could gain energy continuously by degrading organic acids for cell growth and metabolism. And the redox balance was maintained by releasing electrons to the anode, which replaces insoluble metals as the final electron accepter (Lin et al., 2018; Li et al., 2019; Wang et al., 2019).

The second way is also derived from electron transfer. As electrons transferred from cathodes into an intracellular environment, along with the cotransport of protons, the hypothetical mechanism of ATP generation is that the released proton will promote the formation of proton motive force (PMF) and drive ATP synthesis (Rose and Regan, 2015).

Compared with aerobic respiration, the energy generated by oxidative phosphorylation is not enough for cell growth at high rates under anaerobic conditions. The main reason is that due to the low supply level of intracellular ATP, as the intermediate metabolites acted as electron acceptors they were not matched by oxygen levels (Unden and Bongaerts, 1997). Generally, as synthesis of the cell appendages is an energy consuming process, an electron shuttle can be used for supplying more energy through highly effective electron transfer under the same conditions (Liu et al., 2012). In some cases, bacteria can gain enough energy to maintain cell growth and metabolism in MEC systems, even though they do not have a complete native electron transfer chain (Smith et al., 2015).

### Co-enzyme Metabolism

The concentration of co-enzymes, NADH or NADPH, represents the level of intracellular reducing power and are involved in many oxidation-reduction reactions. As redox reactions occur inevitably along with electron generation and consumption, the release or capture of electrons in bioelectrochemical systems will disturb the intracellular steady state environment and cause a shift of metabolic flux (Chen et al., 2012).

In electrochemical systems, oxidation and reduction reactions are performed in the anode and cathode chamber, respectively. In MECs, *Geobacter* can catalyze fumarate reduction with the cathode electrode as the sole electron donor, and this reduction was catalyzed by fumarate dehydrogenase (NADH-dependent) (Mahadevan et al., 2006). For electrochemically inactive bacteria, previous studies have also found that the electrons can be transferred from the cathode to ferric iron *via* NADH generation when using ferric citrate as the electron acceptor by *E. coli*, and the whole process can be achieved without any membrane electron transport carriers (Emde et al., 1989). Meanwhile, an analysis of the theory revealed that the intracellular reducing power (NADH) could be enhanced through biological electrolytic synthesis, and the increased concentration of NADH could affect the intracellular reducing and energy reaction (Pandit and Mahadevan, 2011).

Reversely, the efflux of electrons from the anode chamber may create a relative oxidizing intracellular environment. In an MFC system with *Lactococcus lactis*, homolactic fermentation switched to mixed acid fermentation to keep the balance of intracellular reducing power along with the electricity power output (Freguia et al., 2009).

### The Applications of Bioelectrochemical Systems

Based on the effects of bioelectrochemical systems in microbial metabolism, diverse microbial electrochemical technologies were mainly applied to the production of valuable compounds and the generation of power (Fan et al., 2018).

Microbial electrosynthesis is a novel hybrid of biobased and electrochemical approaches to utilize microbial cells to convert dissolved CO_2_ into value-added organic compounds, such as CH_4_ production with *Methanococcus maripaludis* through a self-secreted compounds to promote CO_2_ reduction (Deutzmann et al., 2015), and acetate production with *Sporomusa ovata* using a novel cathode to facilitate direct delivery of CO_2_ to microbes (Bian et al., 2018).

Electro-fermentation (EF) also uses electrochemistry to affect microbial metabolism. The electron transfer in either anodic EF or cathodic EF can regulate the ORP and the NAD^+^/NADH ratio and then affect the intracellular metabolism (Moscoviz et al., 2016). Recently, an anodic electro-fermentation was carried out using *Corynebacterium glutamicum* to produce L-lysine, and the results showed that adoption of anodic electro-fermentation can balance the redox and energy states of *C. glutamicum* and thus improve the anaerobic production of L-lysine (Vassilev et al., 2018). Cathodic electro-fermentation was also performed for simultaneous biogas upgrading and biochemical production, and the highest biogas content [96% (v/v)] and acetate production (358 mg/L) were achieved (Omar et al., 2018).

Photosynthetic MFCs that combined photosynthesis and generation of electric energy, have also gained much attention recently due to their more sustainable energy production than that of non-photosynthetic MFCs (Pillot et al., 2019). The microbes in photosynthetic MFCs usually contain certain specialized light harvesting complexes that function as the units of photosynthesis. These light harvesting units can sustainably convert solar energy into chemical energy, which are then utilized by traditional exoelectrogens to produce electric energy (Rashid et al., 2019). The integration of photosynthesis with MFC technology has opened several neoteric possibilities for sustainable bioenergy generation.

## Future Prospects and Conclusion

All types of microbial electrochemical technologies are based on the energy interchange: chemical energy into electrical energy (MFCs), electric energy into chemical energy (MECs) and solar energy into electrical energy (Photosynthetic MFCs). For all types of bioelectrochemical systems, energy conversion efficiency is the key factor that determines bioelectrochemical system performance, especially the energy conversion step in which electrical energy is involved. Electron transfer plays an important role in this step, which it is implied is a future direction for METs research. The electron’s behavior, intracellular reducing power and energy metabolism in bioelectrochemical systems is of increasing concern in the context of precise regulation of fermentation and degradation. Understanding the mechanism of electron transfer *via* extracellular and intracellular electron transfer chains would extend the future application of bioelectrochemical systems.

## Author Contributions

TZ, JL, and YJ wrote the manuscript. WZ, YF, FX, WD, PW, and MJ provided literature and data. JM contributed to the writing of the manuscript and the overall manuscript design.

## Conflict of Interest

The authors declare that the research was conducted in the absence of any commercial or financial relationships that could be construed as a potential conflict of interest.
